# Chronic Stress in Pregnancy Is Associated with Low Birth Weight: A Meta-Analysis

**DOI:** 10.3390/jcm12247686

**Published:** 2023-12-14

**Authors:** Alkis Matsas, Panagiota Panopoulou, Neofyta Antoniou, Alexandra Bargiota, Alexandros Gryparis, Nikolaos Vrachnis, George Mastorakos, Sophia N. Kalantaridou, Theodoros Panoskaltsis, Nikos F. Vlahos, Georgios Valsamakis

**Affiliations:** 1 Second Department of Obstetrics and Gynecology, Aretaieion University Hospital, Medical School, National and Kapodistrian University of Athens, 76 Vasilisis Sofias Avenue, 11528 Athens, Greece; 2 Third Department of Obstetrics and Gynecology, Attikon University Hospital, Medical School, National and Kapodistrian University of Athens, 1 Rimini Street, Chaidari, 12462 Athens, Greece; 3 Department of Endocrinology and Metabolic Disorders, University Hospital of Larissa, Medical School of Larissa, University of Thessaly, 41334 Larissa, Greece; 4 Department of Hygiene, Epidemiology and Medical Statistics, National and Kapodistrian University of Athens, Goudi, 11527 Athens, Greece

**Keywords:** chronic stress, birth weight, pregnancy, perinatal period, stress questionnaires

## Abstract

Background and objectives: Chronic activation of the stress system has cumulative effects on the body, and it places individuals at risk for adverse health outcomes. Chronic stress has been assessed by health questionnaires in pregnancy. During the perinatal period, mothers experience increased physical and emotional demands. Chronic stress interferes with hormonal functions in mothers and infants. This meta-analysis studies the effect of maternal chronic stress during pregnancy, as assessed by established stress questionnaires, on the birth weight of their full-term infants. Design and methods: According to our criteria and after research collection, we obtained 107 studies and we conducted two types of analyses: a logistic (N = 22,342) and linear regression analysis (N = 7431). Results: Our results show that chronic stress is associated with a statistically significant risk of low birth weight (OR = 1.50, CI 95% = [1.13; 1.99], *p* ≤ 0.02).Conclusions: Increased maternal chronic stress, as assessed by questionnaires, in pregnancy is associated with a low-birth-weight baby. The above meta-analysis indicates that maternal high chronic stress questionnaire scores could be used as a clinical tool in order to assess low-birth-weight risk.

## 1. Introduction

Stress is a normal response to psychological and physical demands, and threats, and that includes mental health states and stressors such as anxiety, depression, racism, coping mechanisms, job strain, lack of social support and domestic violence [1]. The physiologic stress response aims to maintain homeostasis. Indeed, when the demands and threats overwhelm an individual’s ability to respond, negative health outcomes can result [2]. Any physical or psychosocial stimulus can be stressful when it is perceived as threatening to homeostasis and the survival of the organism [3].

Stress is divided into acute and chronic stress. Acute stress is short-lived, and, in most cases, a successful response is achieved [1]. On the contrary, chronic stress persists for a longer period and, in many cases, without resolution to threats or demands [1]. The constant exposure to stress has cumulative effects on the body and places individuals at risk for adverse health outcomes. Chronic activation of the stress system would be expected to increase vulnerability to diet-related visceral adiposity, decrease lean body mass, and induce insulin resistance [4,5].Uncontrollable stress can also lead to melancholic depression, sleep disorder, and a reduction of quality of life [6].

During the perinatal period, mothers face increased physical and emotional demands. The latter associated with depression can affect essential functions related to mothers and infants. Evidence indicates that stress during pregnancy is associated with behaviors and adverse health practices, such as poor nutrition, delayed prenatal care, adherence to medical recommendations, and use of alcohol and cigarettes, which could lead to adverse birth outcomes [7]. Research on pregnancy focuses on mental disorders such as anxiety and depressive disorders following negative life events or experiences. Evidence shows that chronic stressis linked to restricted fetal growth and low birth weight [8].

According to studies, there is evidence for a relationship between stress and poor birth outcomes [9]. Intrauterine growth restriction and low birth weight emerge as theleading causes of neonatal and infant morbidity and of neurodevelopmental impairments [8]. There is public health concern regarding the high rates of prematurity and low birth weight as they are the leading causes of complications such as respiratory, nervous system, gastrointestinal and immune-related problems.

In order to understand the role of stress on perinatal health, it is important to effectively study and measure its characteristics. Stress levels can be measured by stress questionnaires. They measure external stressors which include life events or daily hassles; perceived stress that reflects perceptions of personal stress levels; and enhancers of stress that include anxiety or depression. The information of a questionnaire can be gathered by an interview method or a checklist format that is answered by respondents [2]. All aim to objectively assess stress and relate their score to specific pathological conditions. 

Due to the large prevalence of intrauterine growth retardation and low birth weight in infants, but also the increasing incidence of stress in life, several studies indicate the role of maternal psychological stress in fetal growth. Their results are controversial; while some studies point out a positive association [10], others show no association between these factors [11].

Consequently, the aim of this meta-analysis is to clarify what is the effect of chronic stress of motherson the birth weight of their full-term babies, using questionnaires during pregnancy for the assessment of chronic stress, extracting data from the existing studies.

## 2. Materials and Methods

An extensive search was performed with Pubmed, Medline, and Google Scholar databases using the terms (a) “stress questionnaire” or “chronic stress”, (b) “fetal growth”, (c) ‘birth weight’ or ‘low birth weight’ and (d) ‘pregnancy’. A combination of (a) AND (b) or (a) AND (c) or (a) AND (d) were also used. References through retrieved articles helped to identify relevant articles and contact prominent investigators in the field. The main outcome was the effect of mothers’ chronic stress on the low birth weight of full-term .neonates.

This review is not registered in the international prospective register of systematic reviews (PROSPERO).

Inclusion criteria: Published English-language longitudinal studies were included. Chronic stress might be assessed by any kind of a valid questionnaire on chronic stress during pregnancy. Newborns might be full-terms and the data might be measured after birth or collected from medical history. 

Exclusion criteria: Studies were excluded if they did not report sufficient data to calculate the relationship between chronic stress and birth weight. Studies were excluded if the participants answered the questionnaires before or after pregnancy. Studies were excluded if they did not deal with valid and explicitly referenced questionnaires on chronic stress. Using these combinations of words and criteria, we obtained 107 studies, and 12 published studies met the inclusion criteria (a total of 29,773 participants). Evaluation of covariables contributing to low birthweight is not equivalent in all included studies.

**Figure 1:**
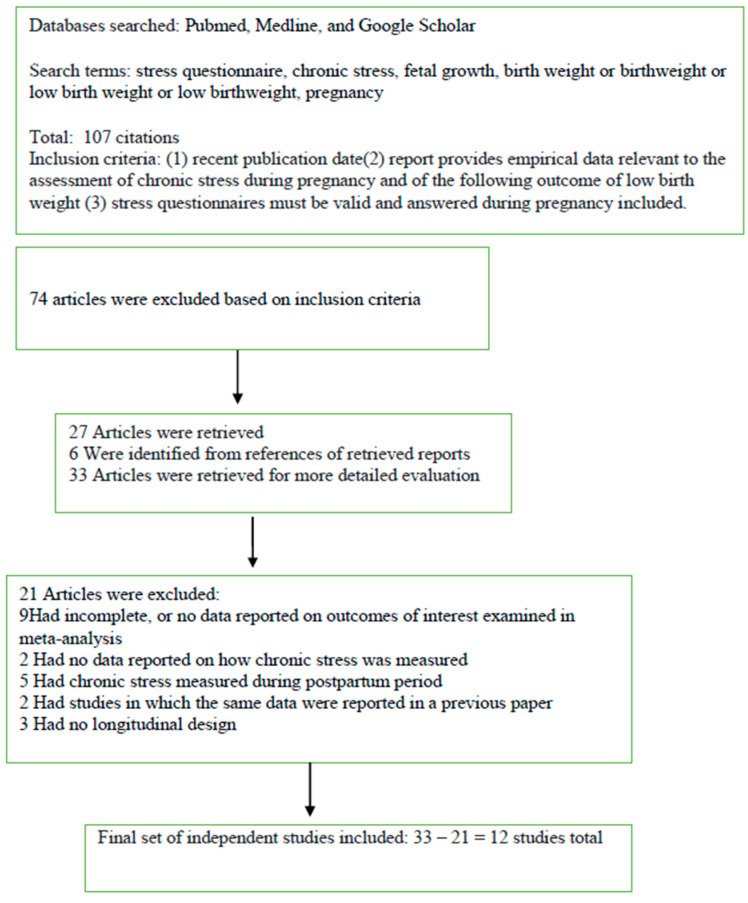
Preferred reporting items for systematic reviews and meta-analysis (PRISMA).

Selection strategies are illustrated in Figure 1. 

After research collection, we conducted two types of analysis: a linear and a logistic regression analysis depending on the analysis of each group of studies. 

Study characteristics

We extracted information from each study using the authors’ definition of clinical significance. Typically, low birth weight (LBW) was defined as less than 2500 g in full-term babies and chronic stress was assessed by valid questionnaires that measured quality of life, perceived stress, and consequences of chronic stress as depression during pregnancy, as presented in Table 1.

In logistic analysis, as shown in Table 2, we concluded Andersson et al.’ study included 1465 women during the 2nd trimester of their pregnancy where they answered Primary Care Evaluation of Mental Disorders (PRIME-MD), a questionnaire that measures depression, anxiety, alcohol, somatoform, and eating disorders. Data about birth weight were recorded from the pediatric medical charts. There was no significant association between antenatal anxiety disorder and the neonatal outcome of birth weight. Odds ratios for this study were adjusted for age, marital status, socioeconomic status, smoking habits, and body mass index [22]. Sgegda et al. conducted a cohort study with 1267 pregnant women. They answered the Perceived Stress Scale (PSS) during pregnancy. Birth weight was abstracted from the medical record and used to classify low-birth-weight infants (<2500 g at birth). A positive association was observed between mid-pregnancy stress and low birth weight, with women in the highest quartile experiencing more than three times the risk of low birth weight compared with women in the lowest quartile of stress adjusted for maternal age, BMI, parity, history of preterm birth (PTB), smoking, anxiety, and probable major depression [10].

Lau recruited a study with 581 pregnant women who answered PSS during the 2nd trimester of pregnancy. Low birth weight was defined as an infant weighing less than 2500 g. Data were obtained from hospital records. They found that those participants with higher perceived stress levels had more risk to have low-birth-weight infants adjusted for demographic and socioeconomic characteristics [23].

Rondo et al. included 865 women. Maternal psychological distress was studied using the General Health Questionnaire (GHQ) during pregnancy. Information about birth weight was obtained by women’s medical history. LBW was a birth weight of <2500 g. This study confirmed that LBW is associated with distress even after controlling for other risk factors [24].

In Evans et al.’s study, 10,967 pregnant women completed the Edinburgh Postnatal Depression Scale (EPDS) at 18 weeks gestation. Birth weight was obtained directly by the research staff. They found that women with a high depressive symptom score were more at risk to give birth to babies with low birth weight, but this was attenuated after adjustment for confounders such as gender, maternal age, gestation, and smoking. Hence, they conclude that there is little evidence of an independent association between depressive symptoms during pregnancy and birth weight [25].

In Berle et al.’s study, 680 women rated themselves on the Hospital Anxiety and Depression Rating Scale (HADS). The outcome variable was birth weight. After adjustment for demographic, obstetric and somatic variables, they concluded that anxiety disorder and depression during pregnancy are not significantly associated with low birth weight [26].

Krabbendam et al. conducted a cohort study with 5511 pregnant women which answered PSS. They included small-for-gestational-age (SGA) babies who were defined with a birth weight less than the 10th percentile for gestational age. The authors found that a high level of perceived stress at 14 weeks of pregnancy increases the risk for delivery of an SGA infant. This association was reduced after adjustment for the possible confounding effects of demographic variables. So, the results did not support a direct relationship between perceived stress and SGA [27]. Borders et al. recruited 294 women in a cohort study that answered the Center for Epidemiological Studies Depression Scale (CES-D). LBW was defined as less than 2500 g and data were obtained through the medical record. In this analysis, they conclude that psychological stress was associated with the delivery of a low-birth-weight neonate even after adjusting for maternal age [28].

In Steer et al.’s study, 712 pregnant women answered the Beck Depression Inventory (BDI) during 28 weeks of gestation. LBW was the main outcome. They found that there was no association between BDI scores with this pregnancy outcome after adjustment for African American ethnicity, low pre-pregnancy, body mass index (BMI), a prior history of LBW delivery, inadequate weight gain, smoking and parity [29].

In linear analysis, as shown in Table 3, we concluded for Henrichs et al.’s study that they recruited 6313 pregnant women in mid-pregnancy where they answered the Brief Symptom Inventory (BSI). Birth weight was obtained from medical records. In this study, lower birth weight was related only to maternal anxiety symptoms during pregnancy. The adjustment was for gestational age in mid- or late pregnancy or at birth, maternal age, height, body mass index, education, ethnicity, smoking during pregnancy, parity, hypertension in pregnancy, gestational diabetes, fetal sex and pre-eclampsia [30].

Broekman et al. included 946 women in the 2nd trimester of their pregnancy who answered BDI. Birth history data were obtained from documented medical record booklets. No association between chronic stress measured by BDI and LBW was found after adjustment for maternal age at the time of birth, household income, ethnicity, hypertension and diabetes history during pregnancy, cigarette smoking during pregnancy, alcohol use during pregnancy, and maternal height [31].

In Dancause et al.’s study, 172 pregnant women answered GHQ. Data about birth weight were obtained from medical records. Based on the final regression model, higher chronic stress levels predicted lower birth weights, with mid-pregnancy exposure having the greatest impact after the control of their results [32].

## 3. Results

Data were analyzed with statistical package of SPSS 12.0 version for Windows (SPSS Inc., Chicago, IL, USA) to explore the relationship between the variable of low birth weight and the effect of chronic stress. We used adjusted odds ratios (ORs) with 95% confidence intervals (CIs) based on demographic and psychosocial variables. The Breslow–Day test (SPSS 12.0 for Windows) was used to evaluate the homogeneity of odds ratios after stratification. 

However, this meta-analysis included two types of analysis: a linear and logistic regression analysis. Nine of them were included in logistics and three of them were included in linear analysis. The purpose of this meta-analysis was to clarify what is the effect of chronic stress on birth weight according to stress questionnaires. 

On the one hand, regarding logistic regression, four of nine studies showed no association between low birth weight and the scores of questionnaires that measure chronic stress [11,26,27,29]. On the contrary, five of nine studies indicated that as chronic stress rises, the effect of LBW increases [10,23,24,25,28].

**Figure 2:**
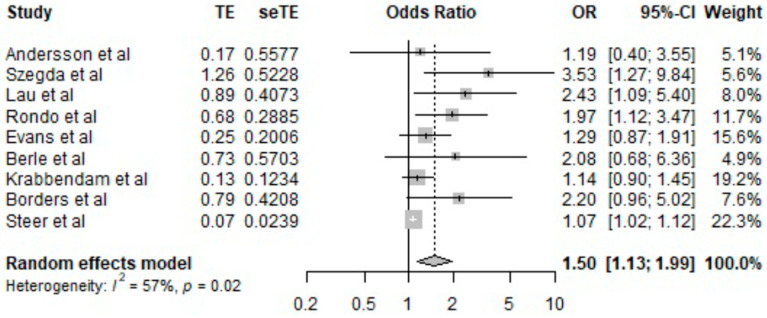
Results of logistic regression analysis [10,11,23,24,25,26,27,28,29].

The association between chronic stress and LBW, after the analysis, is shown in Figure 2. The odds ratios that we used were adjusted. Afterward, the meta-analysis showed that chronic stress is associated with a statistically significant risk of LBW (OR = 1.50, CI 95% = [1.13; 1.99], *p* ≤ 0.02). The heterogeneity of sources was I^2^ = 57%. 

**Figure 3:**
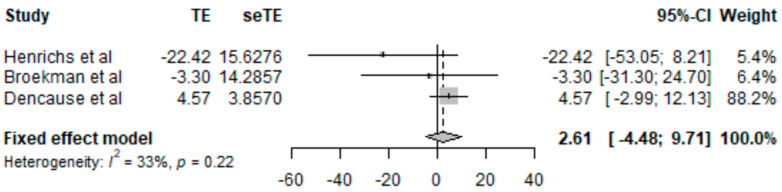
Results of linear regression analysis [30,31,32].

On the other hand, three studies were included in the linear regression analysis as shown in Figure 3. Two of them showed a negative relationship between chronic stress and LBW [30,32]. The heterogeneity was I^2^ = 33% and *p* = 0.22.

## 4. Discussion and Conclusions

This meta-analysis aimed to examine the association between maternal chronic stress using stress questionnaires at pregnancy and birthweight or low birth weight of full-term infants. Stress questionnaires included in studies were valid to be used in the general population or during pregnancy. These questionnaires measure general health status, anxiety, perceived stress, and depression as a consequence of chronic stress. Psychologists have designed several instruments intended to estimate the magnitude of stress rather than measuring stress hormones because the hypothalamus–pituitary–adrenal (HPA) system has a sensitivity to environmental factors such as daytime, the method of sample collection, negative feedback, and the pulsative fashion of hormones that may give false results.

According to birth weight as a prediction of intrauterine fetal growth, we must mention that this is only a final measure of a long, rapid, and non-linear period of intrauterine growth. While undergoing fetal growth restriction due to environmental influences, an individual fetus could still reach a normal birth weight because of its high genetic growth potential [30]. At the same time, the effect of stress on birth weight is dependent on genetic components and the individual’s unique stress response and life course. However, chronic stressors are robust predictors of low birth weight, that is, infants weighing less than 2500 g at birth. Studies have shown that a significant proportion of low-birth-weight infants are preterm births [33]. In addition, the relationship between psychosocial stress and low birth weight may be related to variation in energetic intake and expenditure. For example, pregnant women who run a household without the support of a husband, partner or others may suffer inadequate nutritional provisioning and a greater workload, reducing maternal and fetal weight gain. Depression and chronic strain seem to be stronger predictors of low birth weight [34].

Maternal stress and distress can lead to an elevated maternal HPA axis activity causing an increased release of glucocorticoids which negatively affect fetal development [35]. Maternal stress hormones may be transferred to the fetus by trans-placental passage and by the stress-induced release of placental hormones that enter the fetal circulation. Glucocorticoids have a role in fetal tissue proliferation and differentiation, and are growth-inhibiting [36]. First, maternal stress leads to the release of catecholamines, which can reduce uterine perfusion, potentially limiting the amount of substrate delivered to the fetus; thus, after prolonged exposure to catecholamines, this could contribute to reduced fetal growth. Second, perceived stress during pregnancy may affect appetite, food frequency patterns, and the timing of weight gain, all of which play an important role in fetal growth. Third, stress can elevate endogenous cortisol levels during pregnancy and this may also inhibit fetal growth [34].

The placental enzyme 11beta-hydroxysteroid dehydrogenase type 2 (11ß-HSD-2) acts by inactivating approximately 80–90% of the maternal cortisol [37]. This mechanism is believed to protect the fetus from excessive maternal cortisol concentrations under physiological conditions. In addition, during acute maternal stress situations, it may lead to the greater exposure of the fetus to maternal released cortisol, so the placental barrier is overwhelmed by psychosocial stress, thus exposing the fetus to disproportionately high fluctuations in maternal plasma cortisol. It has been described that prenatal stress itself—or maternal anxiety as a marker of prenatal stress—reduces the expression and activity of 11ß-HSD-2 in humans and animal models [38]. This meta-analysis shows that maternal psychological distress of chronic stress measured by questionnaires can reduce birth weight. However, the main limitation of this review is the small number of included studies, reflecting the statistical analysis. That is why we excluded several studies with missing statistical parameters from this review, beyond the exclusion criteria. The limitation of this meta-analysis is that there are no questionnaires assessing all aspects of chronic stress. Often, studies have difficulties in clarifying differences in symptoms between anxiety and depression, and so we had to include aspects of mental health such as anxiety and depression which are part of chronic stress. Another limitation is that we included studies assessing chronic stress regardless of the trimester of pregnancy they were assessed. Furthermore, we took into consideration that chronic stress is more a stable characteristic, not specific to any set of circumstances or events [39]. Chronic stress is characterized by a stable behavioral and emotional response that is essentially part of the individual’s personality. These are reflected in risky behaviors and adverse health practices, and the adverse health outcomes of chronic stress, which are parameters that we tried to collect from studies and questionnaires.

In conclusion, pregnancies with a high score in questionnaires assessing chronic stress are associated with low-birth-weight neonates. Stress hormones and the cascade of the HPA axis during pregnancy, low weight gaining that characterized stressed women, and preterm birth can be important factors in this relation. A direction for future research may be to further understand the pathophysiology of distress in low birth weight, create more specialized stress questionnaires in pregnancy, and strategies to effectively reduce anxiety and stress.

## Figures and Tables

**Table 1:** Chronic stress questionnaires.

Name of Scale	Description of Scale
Primary Care Evaluation of Mental Disorders (PRIME-MD) [12]	A 2-stage system: the patient first completes a 26-item self-administered questionnaire that screens for 5 of the most common groups of disorders in primary care: anxiety depressive, somatoform, alcohol and eating disorders.
Hospital Anxiety and Depression Rating Scale (HADS) [13]	A total of 14 items developed for the hospital setting: 7 items for symptoms of depression (HADS-D) and 7 items for symptoms of anxiety (HADS-A) experienced during the previous 7 days.
Center for Epidemiological Studies Depression Scale (CES-D) [14]	A total of 20 items which measure symptoms of depression and their frequencies in the past week in the general population; cutoff for depression is a score of 16.
SF-36 Health Survey [15]	Indicates the health status of populations, helps with service planning and measures the impact of clinical and social interventions.
Perceived Stress Scale (PSS) [16]	A total of 14 items measure the degree to which lives are unpredictable, uncontrollable, and overloading in the last 30 days.
Edinburgh Postnatal Depression Scale (EPDS) [17]	A total of 10 items initially developed for measuring the severity of postpartum depression symptoms, which have been validated for use in non-postpartum women.
State—Trait Anxiety Inventories, (STAI) [18]	The 20-item scale measures state or transitory anxiety experienced at that moment only and the other 20-item scale measures trait anxiety, or propensity towards anxiety based on personality.
General Health Questionnaire (GHQ) [19]	Screening tool for detecting psychiatric illness through items regarding disruptions in performing daily activities and feelings of subjective distress; 12-, 28-, 30-, and 60-item versions.
Beck Depression Inventory (BDI) [20]	A total of 21 items that measure the severity of behavioral manifestations of depression, including mood, pessimism, sense of failure, lack of satisfaction, guilt, self-dislike, punishment, self- accusation, crying, irritability, suicidal ideation social withdrawal, indecisiveness, body image, work, sleep disturbance, fatigue, loss of appetite, weight loss, somatic symptoms, and libido.
Brief Symptom Inventory (BSI) [21]	A self-report questionnaire with 53 items that assesses anxious and depressive symptoms. These items define a spectrum of psychiatric symptoms in the preceding seven days.

**Table 2:** Characteristics of studies included in logistic regression.

Source	Sample Size	Questionnaire	OR (95% CI)
[11]	1465	PRIME-MD	1.19 (0.40, 3.55)
[10]	1267	PSS	3.53 (1.27, 9.84)
[23]	581	PSS	2.43 (1.09, 5.40)
[24]	865	GHQ	1.97 (1.12, 3.47)
[25]	10,967	EPDS	1.29 (0.87, 1.91)
[26]	680	HADS	2.08 (0.68, 6.36)
[27]	5511	PSS	1.14 (0.90, 1.45)
[28]	294	CES-D	2.20 (0.96, 5.02)
[29]	712	BDI	1.07 (1.02, 1.12)
[11]	1465	PRIME-MD	1.19 (0.40, 3.55)
[10]	1267	PSS	3.53 (1.27, 9.84)
[23]	581	PSS	2.43 (1.09, 5.40)
[24]	865	GHQ	1.97 (1.12, 3.47)
[25]	10,967	EPDS	1.29 (0.87, 1.91)
[26]	680	HADS	2.08 (0.68, 6.36)

Abbreviations: PRIME-MD = Primary Care Evaluation of Mental Disorders; PSS = Perceived Stress Scale; GHQ = General Health Questionnaire; EPDS = Edinburgh Postnatal Depression Scale; HADS = Hospital Anxiety and Depression Rating Scale; CES-D = Center for Epidemiological Studies Depression Scale; BDI = Beck Depression Inventory.

**Table 3:** Characteristics of studies included in linear regression.

Source	Sample Size	Questionnaire	OR (95% CI)
[30]	6313	BSI	−22.42 (−53.05; 8.21)
[31]	946	BDI	−3.30 (−31.30; 24.70)
[32]	172	GHQ	4.57 (2.99; 12.13)

Abbreviations: BSI = Brief Symptom Inventory; BDI = Beck Depression Inventory; GHQ = General Health Questionnaire.

## Data Availability

Data used in this study are presented within the manuscript.
